# Green synthesis of biomass-derived carbon quantum dots for photocatalytic degradation of methylene blue

**DOI:** 10.3762/bjnano.15.63

**Published:** 2024-06-25

**Authors:** Dalia Chávez-García, Mario Guzman, Viridiana Sanchez, Rubén D Cadena-Nava

**Affiliations:** 1 Centro de Enseñanza Técnica y Superior (CETYS), Camino Microondas Trinidad KM 1, Las Palmas 3era. Sección., 22860, Ensenada, Baja California, Mexico; 2 Centro de Nanociencias y Nanotecnología (CNYN), Ensenada, Baja California, Mexico

**Keywords:** biomass, carbon dots, catalysis, methylene blue, photoluminiscence

## Abstract

Water pollution, significantly influenced by the discharge of synthetic dyes from industries, such as textiles, poses a persistent global threat to human health. Among these dyes, methylene blue, particularly prevalent in the textile sector, exacerbates this issue. This study introduces an innovative approach to mitigate water pollution through the synthesis of nanomaterials using biomass-derived carbon quantum dots (CQDs) from grape pomace and watermelon peel. Utilizing the hydrothermal method at temperatures between 80 and 160 °C over periods ranging from 1 to 24 h, CQDs were successfully synthesized. A comprehensive characterization of the CQDs was performed using UV–visible spectroscopy, Fourier-transform infrared spectroscopy, dynamic light scattering, Raman spectroscopy, and luminescence spectroscopy, confirming their high quality. The photocatalytic activity of the CQDs in degrading methylene blue was evaluated under both sunlight and incandescent light irradiation, with measurements taken at 20 min intervals over a 2 h period. The CQDs, with sizes ranging from 1–10 nm, demonstrated notable optical properties, including upconversion and down-conversion luminescence. The results revealed effective photocatalytic degradation of methylene blue under sunlight, highlighting the potential for scalable production of these cost-effective catalytic nanomaterials for synthetic dye degradation.

## Introduction

The textile industry is known for its high consumption of water, energy, and chemical reagents. For example, manufacturing a pair of indigo-dyed pants requires at least 42 L of water, plus approximately 21 L each time they are washed at home. To ensure product quality, textile dyes are designed to be resistant to light, detergents, and cleaning products, making them difficult to remove. When these dyes are released into water bodies, they cause pollution problems such as chemical oxygen demand, toxicity, and reduced light penetration, which affects aquatic life. As a result, the textile industry is a major contributor to water pollution [1–2].

To address this issue, the textile industry must adopt more sustainable practices in water and energy use, develop environmentally friendly dyes, and implement wastewater treatment processes. Conventional wastewater treatment techniques are often ineffective against resistant dyes, necessitating more advanced and sustainable technologies [3–7]. Embracing more sustainable practices and developing eco-friendly technologies are key to achieving a balance between textile production and environmental preservation [8–14]. Researchers are exploring innovative approaches, such as advanced oxidation processes, nanotechnology-based methods, and biological treatment systems, which show promise in effectively removing pollutants from wastewater [14–16].

In this research, we focus on the synthesis of carbon quantum dots (CQDs) through a hydrothermal method using biomass from watermelon peels and grape pomace. This method is chosen for its ease of production, low cost, and scalability. We aim to evaluate the potential of CQDs as catalysts in the photocatalytic degradation of methylene blue (MB) dye in industrial wastewater.

Photocatalysis is an environmentally friendly water purification method that uses light-activated catalysts to destroy contaminants, offering an advantage over traditional methods that merely transfer contaminants between phases [17–19]. While some studies on CQDs involve costly equipment, our approach uses green synthesis with biomass, aligning with eco-friendly principles [19–21].

Carbon quantum dots are valued for their easy synthesis, good solubility, photostability, nontoxic properties, and versatile applications. They can be synthesized using various methods, including top-down approaches such as arc discharge and laser ablation, and bottom-up methods such as hydrothermal and microwave synthesis [7,22] Biomass sources for CQD synthesis include eggshells, papaya peel, and lemon peel [23–39].

Applications of CQDs range from sensing and cell imaging to drug delivery, photocatalysis, and energy conversion [26–29]. In this study, biomass from watermelon shell and grape pomace waste is used as the carbon source. The hydrothermal method employing urea, nitric acid, and water is utilized. Samples synthesized with nitric acid demonstrated superior catalytic activity in MB degradation.

Carbon quantum dots typically exhibit luminescence properties, with emission peaks in the blue region between 450 and 500 nm varying based on the solvent used [26–28]. In our research, CQDs showed upconversion emission when excited at 900 nm and down-conversion emission in the blue region. The photocatalytic activity of CQDs, under sunlight irradiation, in MB degradation has been confirmed by various studies [32–38].

In this research, samples synthesized with nitric acid exhibited the best catalytic activity in MB degradation, while those prepared with water as a solvent did not show significant catalytic activity. The samples were also characterized using UV–visible spectra, Fourier-transform infrared spectroscopy (FTIR), Raman spectroscopy, dynamic light scattering (DLS), and photoluminescence spectroscopy (PL). The utilization of biomass as a carbon source offers great opportunities due to its abundance in nature and the wide range of sources available, which exhibit good luminescent and catalytic properties.

Focusing on the hydrothermal synthesis method using biomass-derived precursors, this research aims to develop a more sustainable and cost-effective approach for producing CQDs as catalysts in the photocatalysis process. Utilizing watermelon peels and grape pomace as raw materials contributes to waste valorization and promotes a circular economy concept. The ultimate goal is to provide a greener and more efficient solution for wastewater treatment in the textile industry, reducing environmental impact and conserving clean water resources. This research represents a significant step towards addressing water pollution caused by the textile industry, and highlights the potential of nanotechnology in developing sustainable solutions.

## Experimental

### Materials/material synthesis

The synthesis of CQDs involved the utilization of biomass-derived precursors. The method employed was based on the hydrothermal approach reported by Yuan et al. [40] with modifications in terms of precursor selection and chemical conditions.

Two organic materials, watermelon rind and grape pomace, were used as the precursors and were obtained from waste generated by the local wine industry. Initially, small pieces of the biomass were cut and placed on metal trays, followed by drying in a convection oven at 60 °C. Subsequently, the dried precursors were individually crushed in a mortar until they formed a fine powder. Additionally, four different synthesis schemes were proposed, involving modifications in the chemical conditions of the precursor solution.

In a typical synthesis, 50 mL of deionized water was mixed with 0.5 g of biomass precursor powder (dispersion A) and stirred for 10 min. Then, 1.0 g of urea (hydrolizing agent under hydrothermal conditions) was diluted in 50 mL of deionized water to form a homogeneous solution (solution B) and then mixed with dispersion A. The final mixture was stirred for an additional 10 min. Subsequently, the mixture was transferred to a Teflon-lined stainless steel autoclave, which was sealed under pressure using a manual press. The autoclave was then placed in a convection oven and maintained at 100°C for 2 h. After the specified time had elapsed, the autoclave was allowed to cool and the solution was retrieved. After the synthesis, the solutions were filtered through a Büchner funnel equipped with a 200 nm nylon membrane (Whatman) [30] and centrifuged at 15000 rpm for 20 min.

The process previously described was repeated modifying the chemical condition by varying solution B as follows: i) 1.0 g of urea in 50 mL of deionized water (samples M1 and M5); ii) 1.0 g of urea and 5.0 mL of nitric acid (69% v/v) in 50 mL of deionized water (samples M2 and M6); iii) 2.5 mL of nitric acid (69% v/v) in 50 mL of deionized water (samples M3 and M7); and iv) 50 mL of deionized water (M4 and M8). Samples M1, M2, M3, and M4 were prepared using grape pomace peel as the biomass precursor, and samples M5, M6, M7, and M8 were prepared using watermelon peel as the biomass precursor. This information is sumarized in Table 1.

**Table 1:** Synthesis conditions for CQDs.

Sample	Carbon source	Chemical treatment	Hydrothermal conditions

M1	watermelon peel	urea	90 °C/2 h
M2	watermelon peel	nitric acid	90 °C/2 h
M3	watermelon peel	nitric acid + urea	90 °C/2 h
M4	watermelon peel	pure water	90 °C/2 h
M5	grape pomace	urea	90 °C/2 h
M6	grape pomace	nitric acid	90 °C/2 h
M7	grape pomace	nitric acid + urea	90 °C/2 h
M8	grape pomace	pure water	90 °C/2 h

### Characterizations

To determine the optoelectronic characteristics of the synthesized CQDs, UV–vis absorption spectra were measured using a Thermo Scientific Evolution 220 spectrophotometer. The CQDs were dissolved in deionized water at a ratio of 1:10 (0.3 mg of CQDs per mL) and the measurements were taken in the range of 200–800 nm. A 1 cm path length quartz cell was used for the measurements, and deionized water was used as the blank in the measurement to account for background signals. Fourier-transform infrared spectra were measured on a Spectrum Two FT-IR/Sp 10 S/W spectrometer (USA) with a LiTaO_3_ type detector, the wavelength used ranged from 450 to 4000 cm^−1^. The size distribution of the synthesized CQDs was determined via DLS, which relies on the measurement of the hydrodynamic radius of the particles. The CQDs were analyzed via a Malvern NanoSizer ZP instrument. The samples were diluted in deionized water to prevent signal saturation. Multiple measurements (at least three) were performed for each sample to ensure reliable and accurate measurements. A quartz cell with a 1 cm path length was used for the DLS measurements using a Malvern Zeta-sizer equipment model 7.2. Raman spectroscopy for all samples was performed in a Horiba Jobin Yvon Xplora Raman microscope using a 532 nm laser excitation as the power source. The photoluminescence spectra of the samples were obtained with an Agilent Cary Eclipse fluorescence spectrophotometer. It consists of two Czerny–Turner slits (excitation and emission) with a double monochromator and a continuous emission xenon light source (190–900 nm). All the samples were dispersed in deionized water and analyzed with an excitation wavelength of 350 and 900 nm in aqueous medium.

### Photocatalytic evaluation

The photocatalytic activity of the synthesized CQDs was studied for the photodegradation of MB in aqueous media. The tests were separately conducted using sunlight and incandescent light (tungsten halogen lamp, 40 W). Previous to exposure, the reaction mixture was kept under vigorous stirring in the absence of light for 20 min to discard any adsorption effect. On the other hand, the pH value of the reaction mixture was kept constant during the test (pH 7). To initiate the catalytic process, each CQD sample was individually applied to the degradation of the MB dye. The UV–vis absorption spectrum of the dye was monitored using a spectrophotometer (Thermo Scientific Evolution model 220) with a 1 cm path length quartz cell.

To evaluate the photocatalytic activity of the CQDs, a suspension containing CQDs and MB (10 ppm initial concentration of MB) was placed in a baker under constant stirring. Before initiating the light irradiation, the suspension was stirred for 20 min at 400 rpm in a dark environment to ensure proper dispersion of the CQDs in aqueous media and the adsorption of the dye on the CQD surface. After the stirring period of 20 min, the solution absorbance was measured using a spectrophotometer in the wavelength range of 250–800 nm. This measurement marked the start of the photodegradation reaction under light radiation. In the case of tests conducted with incandescent light, a lamp was used as the light source, placed approximately 10 cm away from the solution, and the reaction was allowed to proceed for 100 min.

During the degradation tests, both sunlight and incandescent light were used, and the solution was continuously agitated. The absorbance of the solution was measured at 20 min intervals to monitor the progress of the degradation reaction. All samples were evaluated under both light sources.

## Results and Discussion

### UV–vis spectroscopy

**Figure 1:**
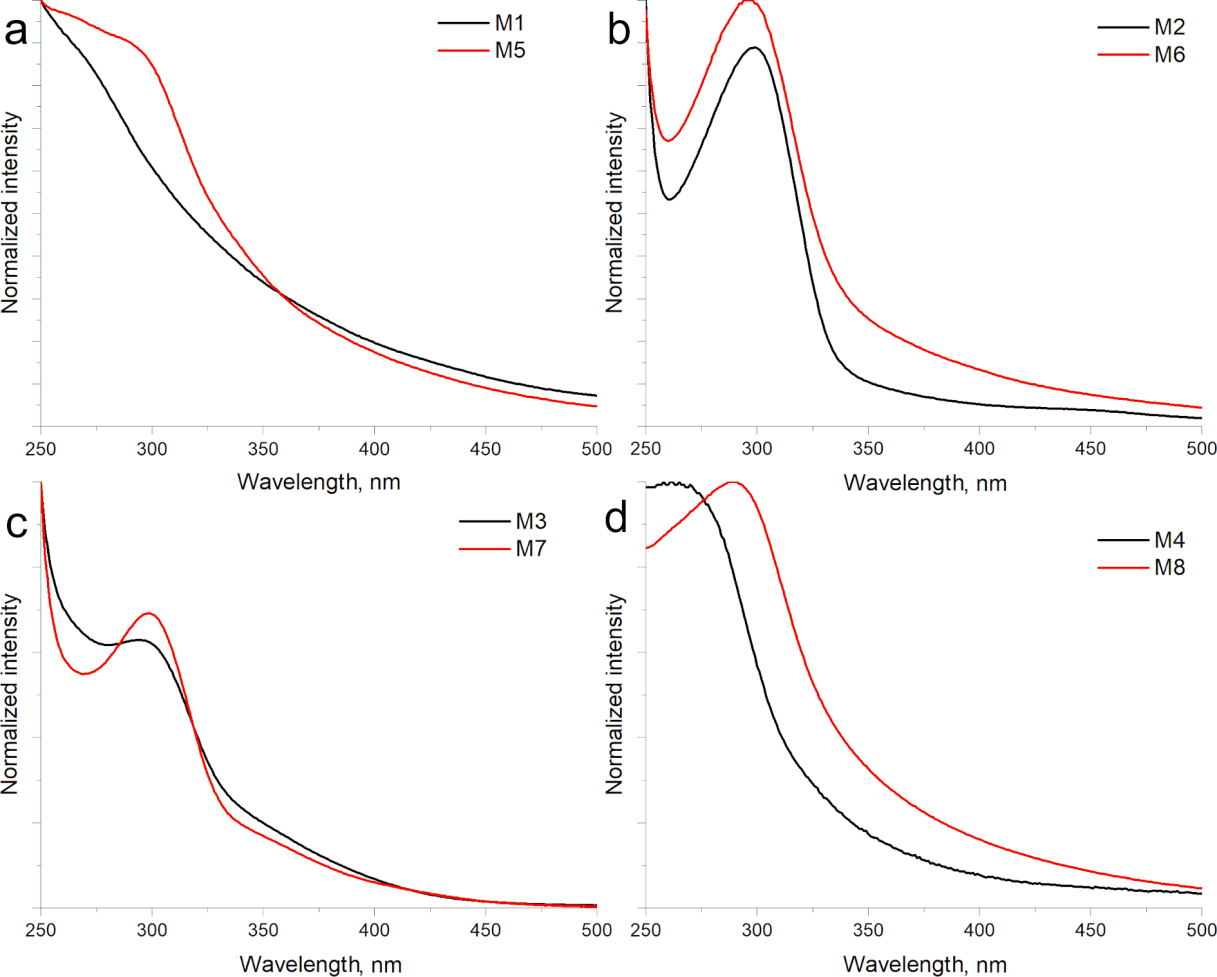
UV–vis absorbance spectra of CQD samples synthesized with a) urea (M1 grape and M5 watermelon); b) urea and nitric acid (M2 grape and M6 watermelon); c) nitric acid (M3 grape and M7 watermelon); and d) deionized water (M4 grape and M8 watermelon).

UV–vis spectroscopy was used to analyze the optoelectronic properties of eight CQD samples synthesized by the hydrothermal method. All samples were diluted in deionized water to adjust the CQDs concentration to 0.3 mg/mL. Figure 1 shows the CQDs synthesized from grape pomace peel (Figure 1a) and watermelon peel (Figure 1b). It is observed that for each of the samples, its maximum absorption peak is approximately at 350 nm, which corresponds to n–π^*^ transitions present in unsaturated compounds with heteroatoms (carbonyl groups). An absorbance tail is also noticed in the visible range [31–32]. The samples M6 and M7 (Figure 1b and Figure 1c) present a relatively symmetrical band also centered at 350 nm with a higher intensity for sample M6. Both samples were prepared using an oxidation process with nitric acid prior to the hydrothermal treatment to break the structure of the carbohydrate used. On the other hand, samples M5 and M8 exhibit an asymmetric behavior in their absorption band centered around 340 nm. The difference can be associated with the polydispersity of the CQDs due to the absence of acid oxidation processes. In this case, the hydrothermal treatment is solely responsible for the formation of CQDs. This same condition was observed when modifying the type of precursor (grape pomace). However, in the case of the samples prepared from grape pomace, a considerably less intense band is observed for the systems prepared with urea (M1 and M5). This is possibly associated with a low production of CQDs (Figure 1a). Samples M1 and M5 exhibit a barely noticeable band between 250 and 300 nm, a typical peak was not observed for both samples probably due to the presence of some carbon impurity that hinder the detection of the π–π^*^ transition around 270 nm.

### Dynamic light scattering

**Figure 2:**
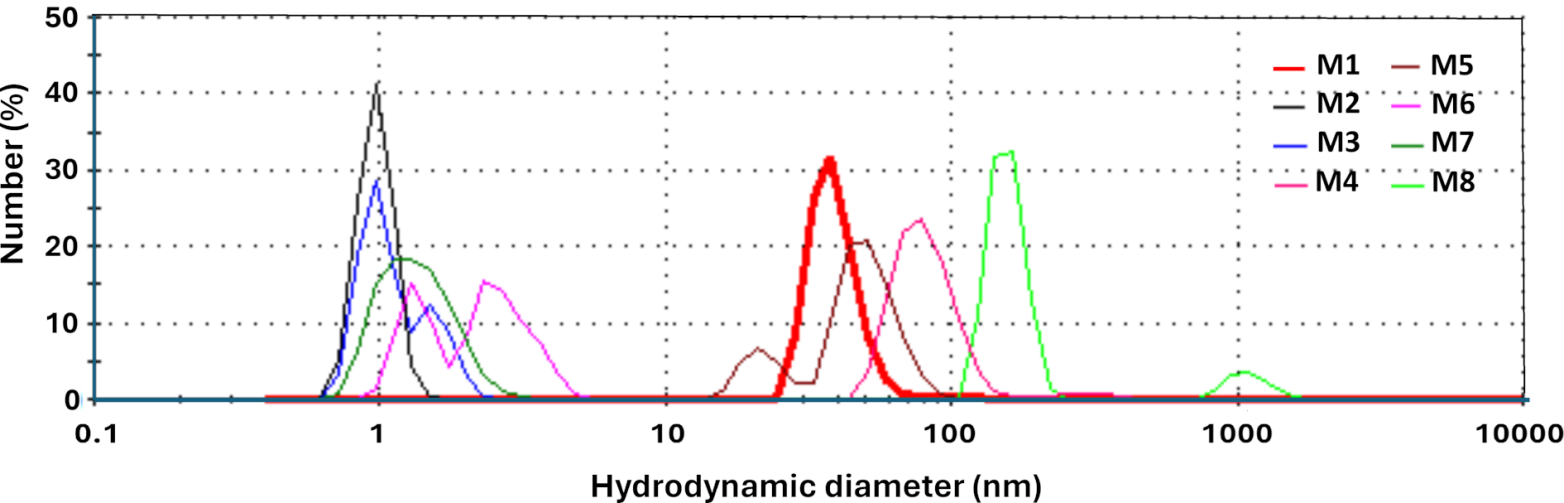
Size distribution of CQDs by dynamic light scattering.

Figure 2 shows the size distribution of the CQDs by DLS. Notably, samples M2, M3, M6, and M7, synthesized with acid, exhibit characteristic sizes of quantum dots within the range of 1 to 10 nm. In contrast, samples M1 and M5, synthesized with urea, display larger sizes ranging from 10 to 100 nm, while samples M4 and M8, synthesized only using water, have sizes close to 100 nm. It is important to note that the samples prepared using grape pomace peel as the biomass source produced smaller particle sizes compared to those prepared with watermelon peel under identical synthesis conditions. The size distribution determined by DLS underlines the influence of biomass source and synthesis parameters on the resulting CQD dimensions.

### Fourier-transform infrared spectroscopy

**Figure 3:**
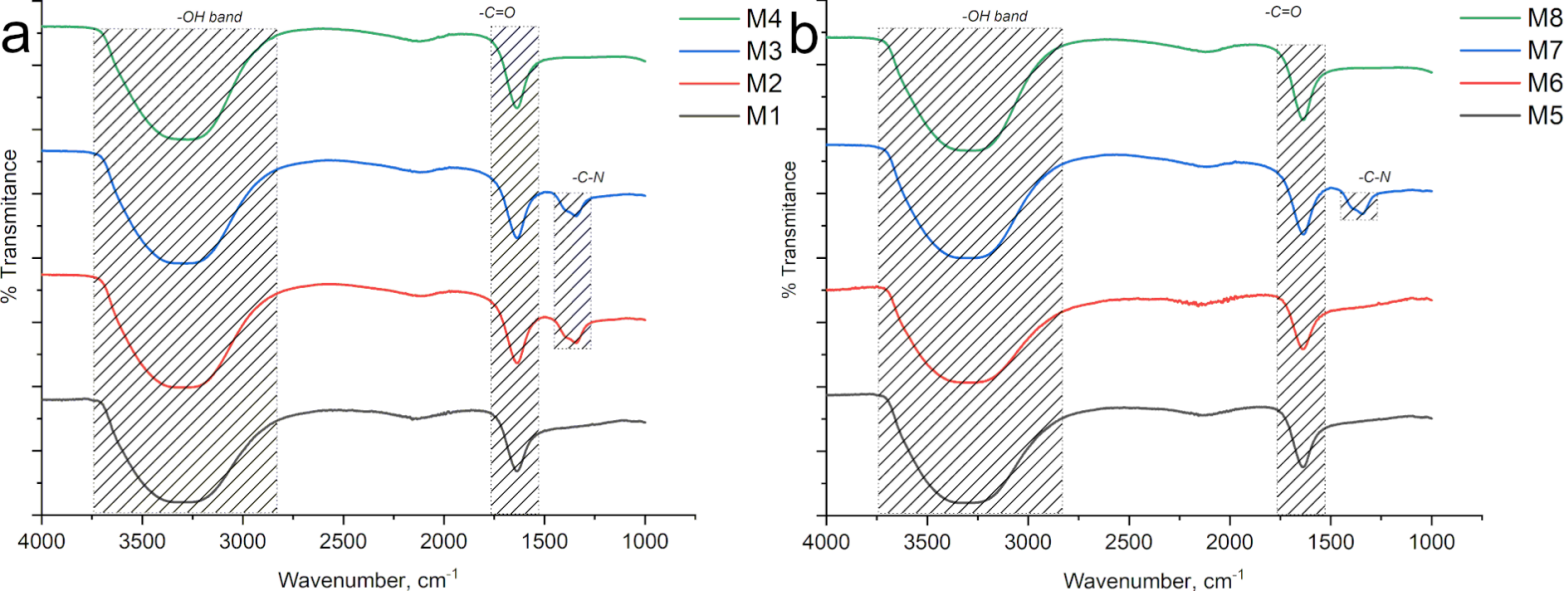
FTIR of a) grape pomace peel with carbonyl peaks, carbon/nitrogen bonds, OH vibrations and some overtones of the benzene ring can be observed, and b) watermelon peel shows characteristics similar to grape pomace samples.

The surface chemical groups of the samples were analyzed using FTIR spectroscopy. Figure 3a displays the FTIR spectrum of the CQDs synthesized with grape pomace. Since the systems were prepared in water, some of the signals may have been shielded. In the spectrum, the absorption bands at 3350 cm^−1^ correspond to OH vibrations and N–H bonds, likely originating from water derived from the oxidation process. These bands are characteristic of the hydroxyl groups present in the acid structure. The peaks around 1600 cm^−1^ fall within the C–O range. The samples synthesized with nitric acid oxidation exhibit an additional peak at 1400 cm^−1^, associated with C–H and C–N bending vibrations, indicating the introduction of nitrogen atoms and oxygen-containing groups. The oxidation process enhances the solubility of CQDs in water. The spectrum also shows low-intensity signals in the range of 1800 to 2500 cm^−1^, which are characteristic of aromatic compounds [22,27].

In Figure 3b, the FTIR spectrum of the CQDs synthesized from watermelon peel is shown. Similar characteristics to the grape pomace CQDs samples can be observed, especially for sample M7, which was also synthesized using nitric acid (similar conditions as sample M3). However, the characteristic carbon/nitrogen groups of the acid, are not observed in sample M7. This could be attributed to the complete consumption of these groups during the treatment, resulting in their signals not being detected in this analysis.

### Raman spectroscopy

**Figure 4:**
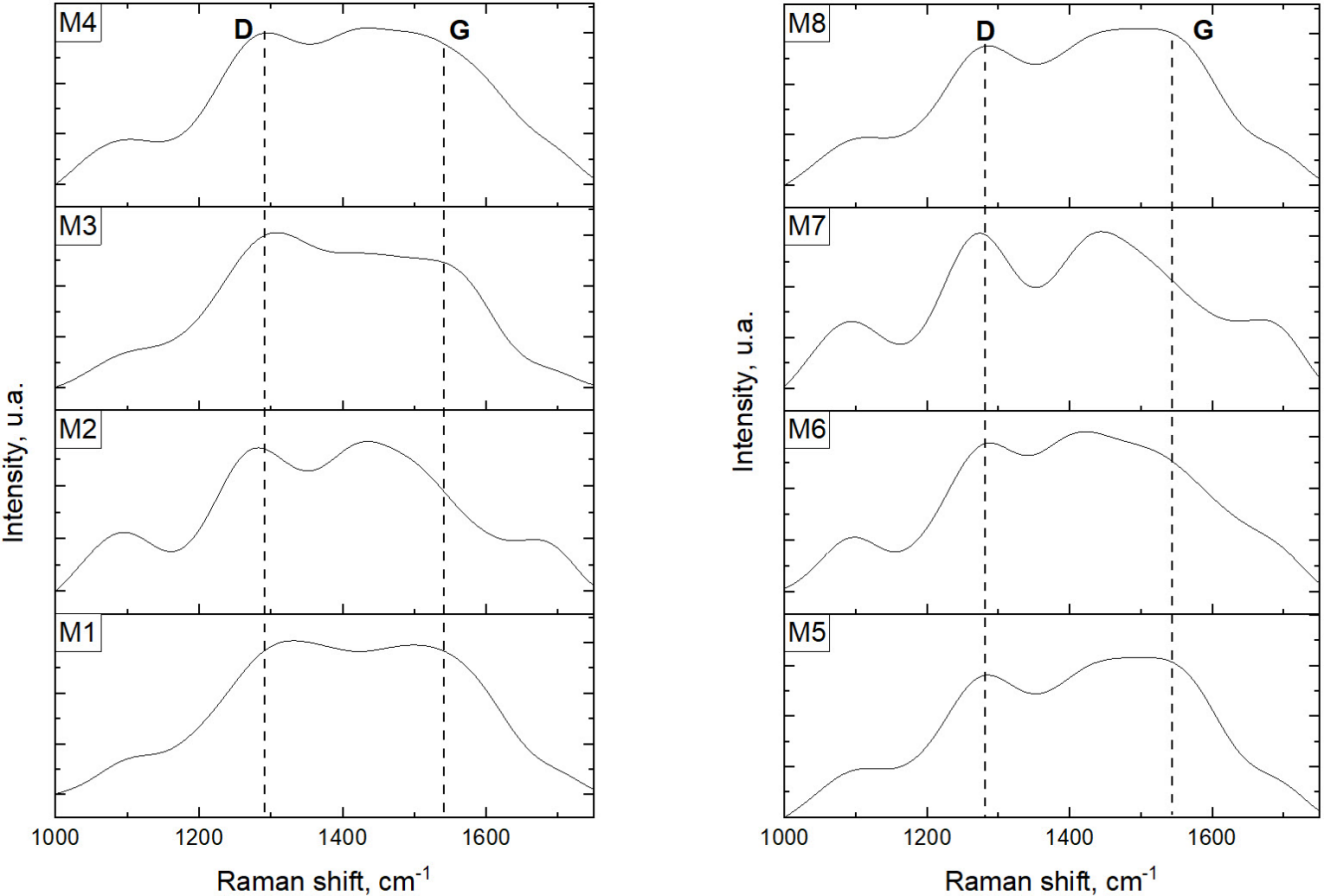
Raman spectra recorded for CQDs prepared from grape pomace (left) and watermelon peel (right) as carbon sources.

Figure 4 shows the Raman spectra for all synthesized samples. On the left side, the spectra of the samples prepared from grape pomace are shown. Two broad bands appear in all samples, one centered around 1300 cm^−1^ and another one centered around 1550 cm^−1^. These features can be related with the D and G bands, respectively [41]. The D band, usually centered around 1385 cm^−1^, corresponds to C with an sp^3^ hybridization and is commonly associated with disordered surfaces. On the other hand, the G band is typically located around 1575 cm^−1^, and corresponds to C with an sp^2^ hybridization which is related with the graphitic structure [41]. Nevertheless, all of our samples presents a noticeable broadening of the peak and a shift in the position of the D and G bands, similarly to what has been previously reported [18,42]. In both cases, such modification in the D and G bands was correlated with the presence of heteroatoms in the CQD structure. The synthesis method reported in this work involves chemical (nitric acid treatment) and physical conditions (hydrothermal treatment) which promotes the inclusion of heteroatoms into the CQDs. All the prepared CQDs samples presented an intensity ratio (*I*_D_/*I*_G_) around 1.0, which corresponds to a poor crystallinity in the CQDs and, as a consequence, a high relative content of defects.

### Photoluminescence spectra

Investigating the underlying origin and mechanisms governing the multi-fluorescence behavior of carbon dots has garnered significant interest in recent times. Diverse research groups have delved into the fluorescence characteristics of CQDs, presenting varied mechanistic explanations. These encompass phenomena such as recombination of electron–hole pairs, quantum effects, surface functional groups, surface states, molecular states, and fluorophores exhibiting differing degrees of π-conjugation. Generally, CQDs comprise a carbon-core domain and surface domains [29].

In the context of PL processes in CQDs, the emission of fluorescence is intriguing and often associated with the presence of surface defects. Various researchers have highlighted the role of radiative recombination of electron–hole pairs and the influence of functional groups within the carbon network in driving the fluorescence phenomenon [24,30–31]. Furthermore, carbon nanomaterials exhibiting fluorescence, such as carbon oxide dots, exhibit a diverse array of structural elements, including sp^2^ carbon hybridization or partial hybridization commonly observed in carbon oxide dots [30–31].

**Figure 5:**
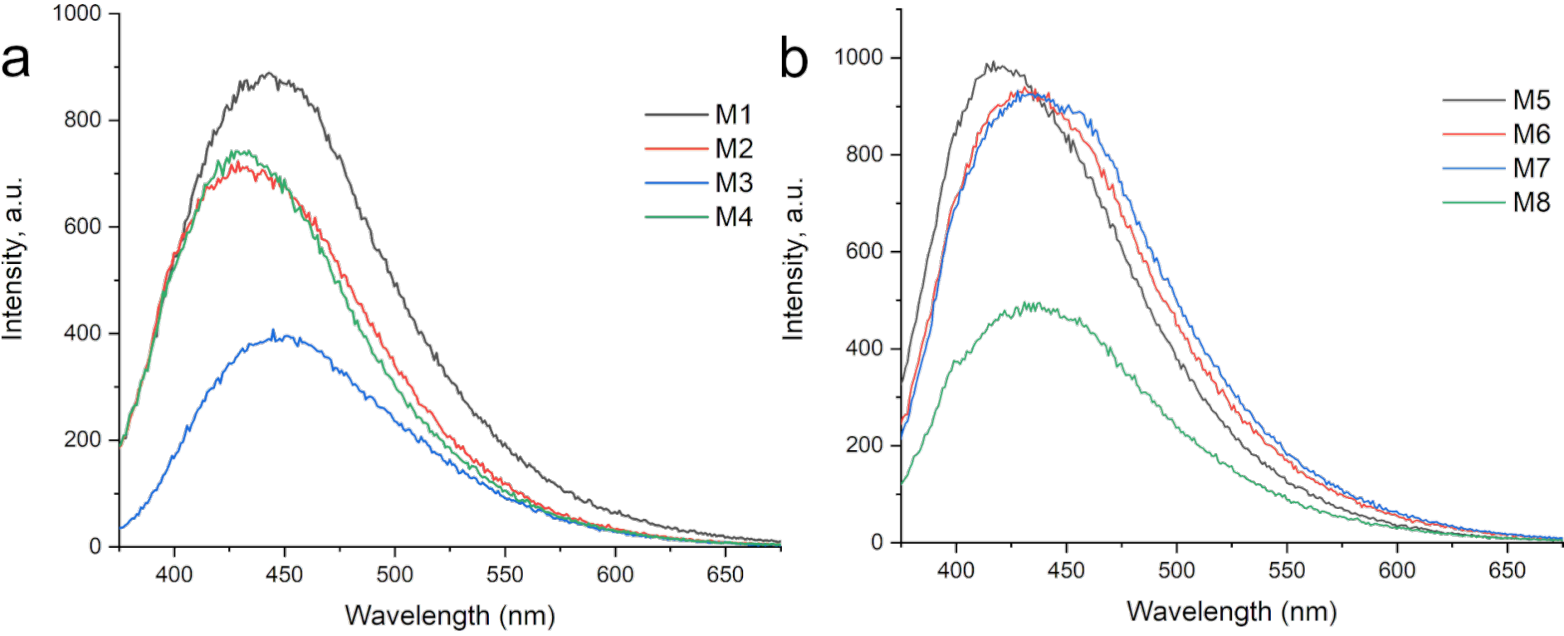
PL spectra, the samples were excited at 350 nm. The graphics corresponds to a) grape pomace peel and b) watermelon peel.

The PL down-conversion spectra of the CQDs synthesized from grape pomace and watermelon peel are shown in Figure 5a and Figure 5b, respectively. All samples were excited at a wavelength of 350 nm, and the emission was observed in the range of 440-450 nm. The samples prepared with urea, namely M1 and M5, exhibited higher intensity in the PL spectra. The PL emission of CQDs can also be influenced by the surrounding acidic conditions, potentially leading to fluorescence quenching. Notably, the sample M3 synthesized using nitric acid demonstrated lower PL intensity, possibly due to such quenching effects under acidic conditions [32].

**Figure 6:**
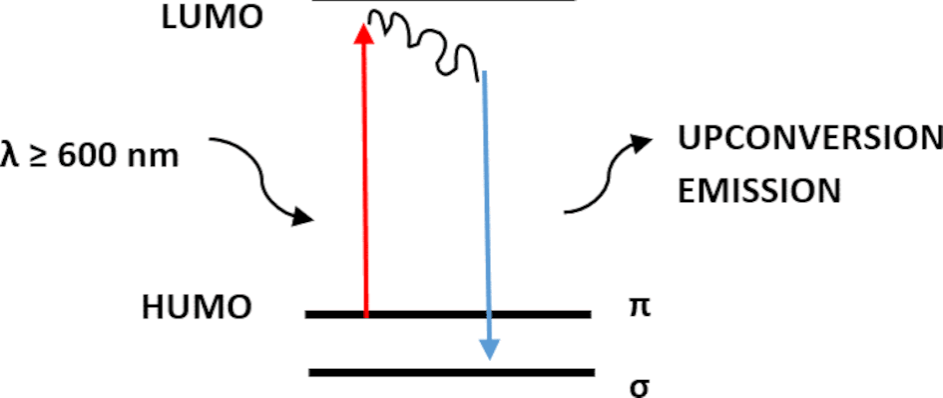
Upconversion process of the quantum confinement effect.

**Figure 7:**
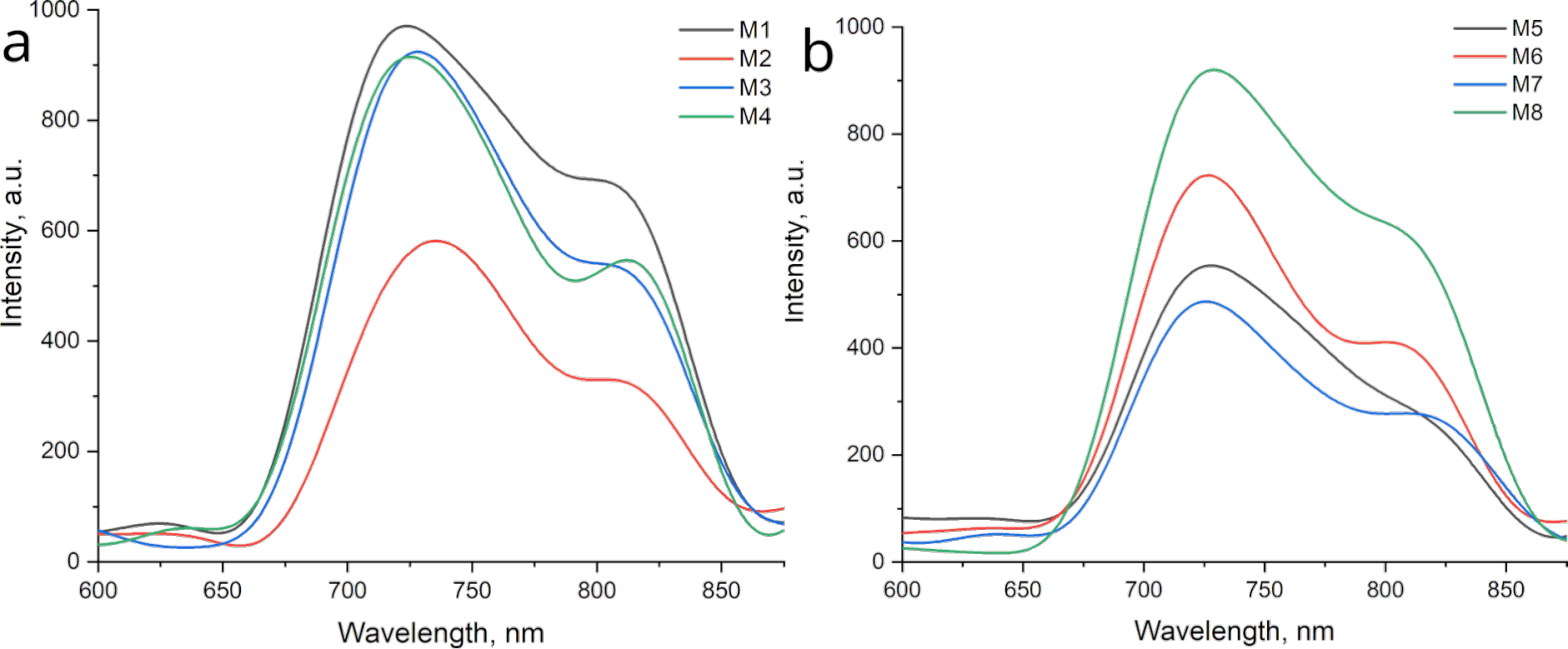
Upconversion PL of the samples, the graphics corresponds to grape pomace peel (M1 to M4) and watermelon peel (M5 to M8). Excitation wavelength: 900 nm.

The upconversion luminescence observed in Figure 7 shows a phenomenon where the emitted fluorescence has a shorter wavelength than the excitation wavelength. This is different from the down-conversion luminescence, where the excitation occurs in the UV region and the emission takes place in the visible spectrum. Unlike down-conversion luminescence, upconversion luminescence does not require high photon density and can occur under normal excitation conditions [31]. Several authors have reported the existence of upconversion carbon dots (CDs). For example, Zhu et al. [43] demonstrated upconversion CDs, while Zhuo et al. [44] reported graphene CDs with upconversion luminescence. The theoretical framework proposed for upconversion photoluminescence centers on the concept of quantum confinement effect (QCE). In this scenario, electrons migrate from the lowest unoccupied molecular orbital (LUMO) to the highest occupied molecular orbital (HOMO) when the excitation wavelength surpasses 600 nm. This process is elucidated in Figure 6 [23,34].

In Figure 7 is observed the upconversion PL of all samples, with excitation at 900 nm and emission in the visible spectra at 720–730 nm. The samples from grape pomace were the ones with more emission than the samples from watermelon, showing similar behavior as the down-conversion process. Besides the main emission band centered around 720–730 nm, all samples exhibited a second band around 840 nm. Considering the upconversion PL mechanism, the authors consider that the presence of heteroatoms in the CQD structure promotes the formation of intermediary states, which may give place to a new emission band. A similar upconversion PL was reported by Wang et al., for CQDs obtained by the hydrothermal method and decorated with BiVO_4_ [45].

### Photocatalytic activity

To carry out the evaluation of the catalytic activity with each of the obtained samples, the curves were first fitted using the calculation of the reaction rate with the pseudo-first-order method. The calculation of the reaction rate constant can be performed using the first-order kinetic model. The first-order rate equation is given by:




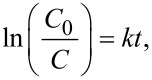




where *C*_0_ is the initial concentration of the reactant (MB dye), *C* is the concentration of the reactant at time *t*, *k* is the first-order rate constant, and *t* is the reaction time.

**Figure 8:**
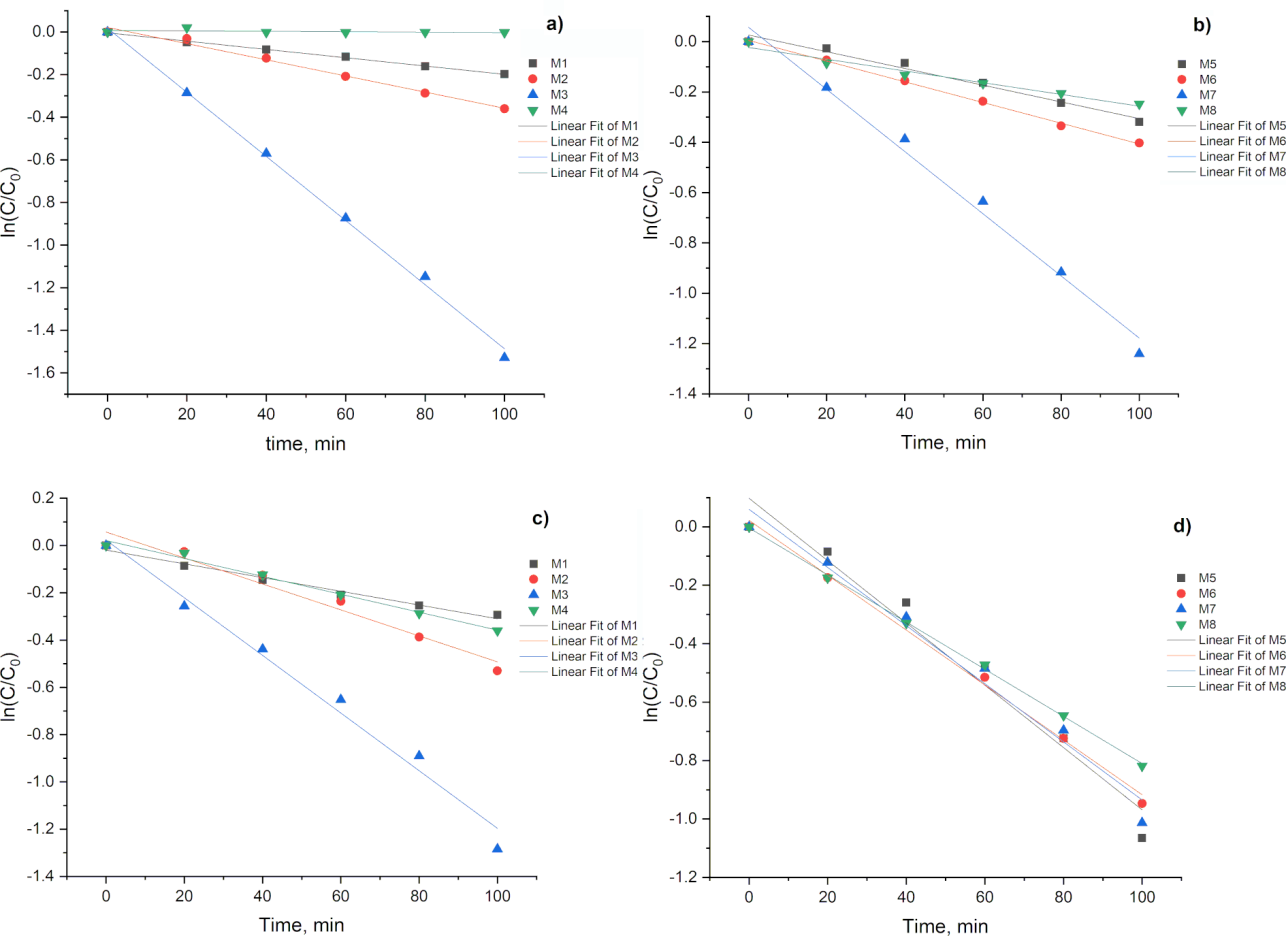
First order adjustment for MB photodegradation for a) grape pomace, W lamp; b) grape pomace, sunlight; c) watermelon peel, W lamp; and d) watermelon peel, sunlight.

The value of the kinetic rate constant (*k*) was estimated based on the slope of the linear fit of the ln(*C*_0_/*C*) as a function of *t* (Figure 8). The concentration of MB was estimated based on the absorbance of the peak centered at 664 nm, using the Lambert–Beer law. As expected, the synthesized CQDs were catalytically active for the photodegradation of MB. All samples displayed a linear behavior and exhibited *R*^2^ values around 0.98 for the degradation of the MB dye (Figure 8), except for the sample M4 under a tungsten 40 W lamp (W lamp), which did not show significant changes on the concentration of the MB. Besides, sample M8 displayed the second lowest rate constant, given that samples M4 and M8 were synthesized in the absence of any hydrolyzing agent (urea) or acidic compound (nitric acid). This led to poor degradation of organic matter, resulting in relatively large carbon structures (over 100 nm) as it was shown in Figure 4, which does not correspond to the size range of a CQD. Nevertheless, when these samples were exposed to sunlight, both exhibited a remarkable increment on their catalytic activity. This indicates that, even when the size on these carbon structures does not correspond to that of the CQDs, the photodegradation can be activated possibly due to an electron–hole pair on the surface of the carbon structure. Due to its size, the crystal structure effects can be enhanced.

The expected mechanism for the photodegradation of MB is related with the formation of electron–hole pairs that, due to their oxidizing properties, promotes the formation of OH^−^ radicals. These species are responsible for the mineralization of the MB molecule [38]. Other aspects that contribute to the MB degradation are the adsorption capacity of MB on the photocatalyst surface (i.e., CQD) and the specific surface area of the CQDs.

All samples (but M3 and M7) presented a similar behavior, increasing their photocatalytic activity when exposed to the sunlight in comparison with their performance under incandescent lamp. It is commonly known that the sunlight emission spectrum presents a higher intensity at the ultraviolet range if compared to that of the tungsten lamp. Considering the band gap of the CQDs, we can affirm that sunlight does create electron–hole pairs more efficiently than the incandescent lamp, which increases the photocatalytic activity of the CQDs.

On the other hand, samples M3 and M7 displayed an opposed behavior, decreasing their photocatalytic activity when exposed to sunlight in comparison to the W lamp. Both samples were prepared under the same conditions but using two different sources of biomass. As nitric acid was used during the synthesis, the presence of C–N groups on the as-synthesized CQDs is expected, which was confirmed by FTIR spectroscopy (Figure 3). According to the report by Rani in 2018 [46], the presence of heteroatoms into the CQDs, such as oxygen, sulfur, and nitrogen, leads to interband states, which decreases the recombination speed of electron–hole pairs. Such delay enhances the photodegradation rate, which was observed for both samples (M3 and M7) independently from the nature of the biomass source.

Considering that the MB molecule contains azo bonds consisting of double bonds (–N=N–), it is possible to foresee a strong interaction with C–N groups on the surface of species M3 and M7. On the other hand, such C–N groups could cause a shift in the electronic energy states [43], which facilitates the formation of electron–hole pairs when irradiated with a W lamp, resulting in a higher photocatalytic activity in comparison to that with sunlight irradiation.

Even when samples M2 and M6 were synthesized using also nitric acid, the formation of C–N groups on their surfaces could be attenuated by the urea (also used during this synthesis), given that above 90ºC urea decomposes forming basic species that can partially neutralize nitric acid acidity.

**Figure 9:**
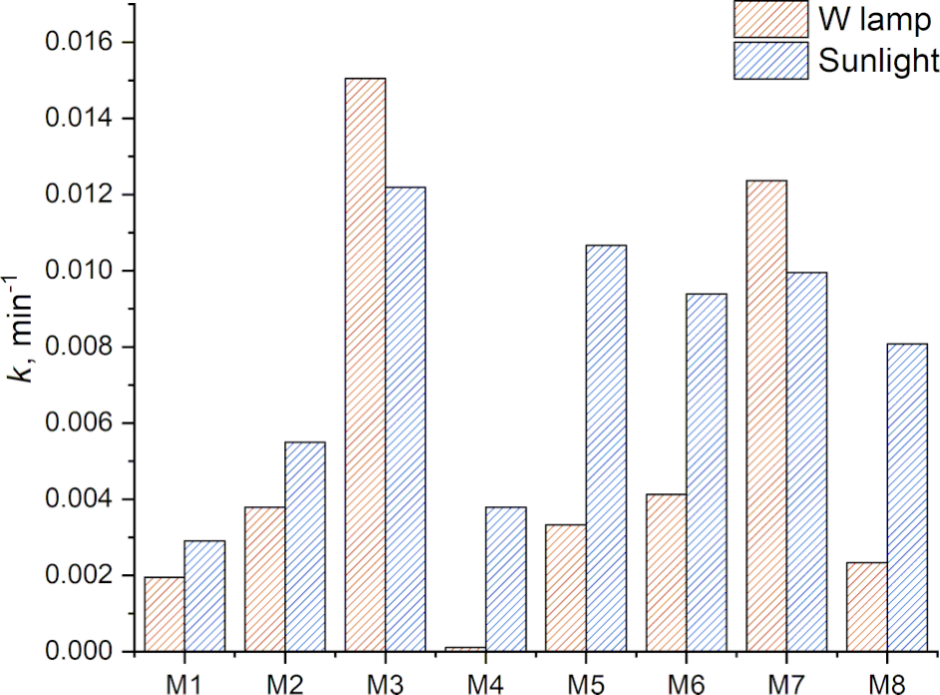
Rate constant values for the photocatalytic activity of CQDs synthesized from grape pomace and watermelon peel. (a) Sunlight radiation in blue, b) light radiation with W lamp in red.

Additionally, the samples with smaller sizes (ranging from 1 to 10 nm) as determined by DLS, display higher catalytic activity. Specifically, samples M4 and M8 synthesized from grape pomace and watermelon, respectively, show considerable catalytic activity under sunlight irradiation. However, when analyzed under incandescent light, these samples exhibit very low activity, likely due to their specific chemical composition and the variations in light intensity between the two light sources. The comparison of the kinetic rate constant (*k*) showing the catalytic activity is shown in Figure 9.

**Figure 10:**
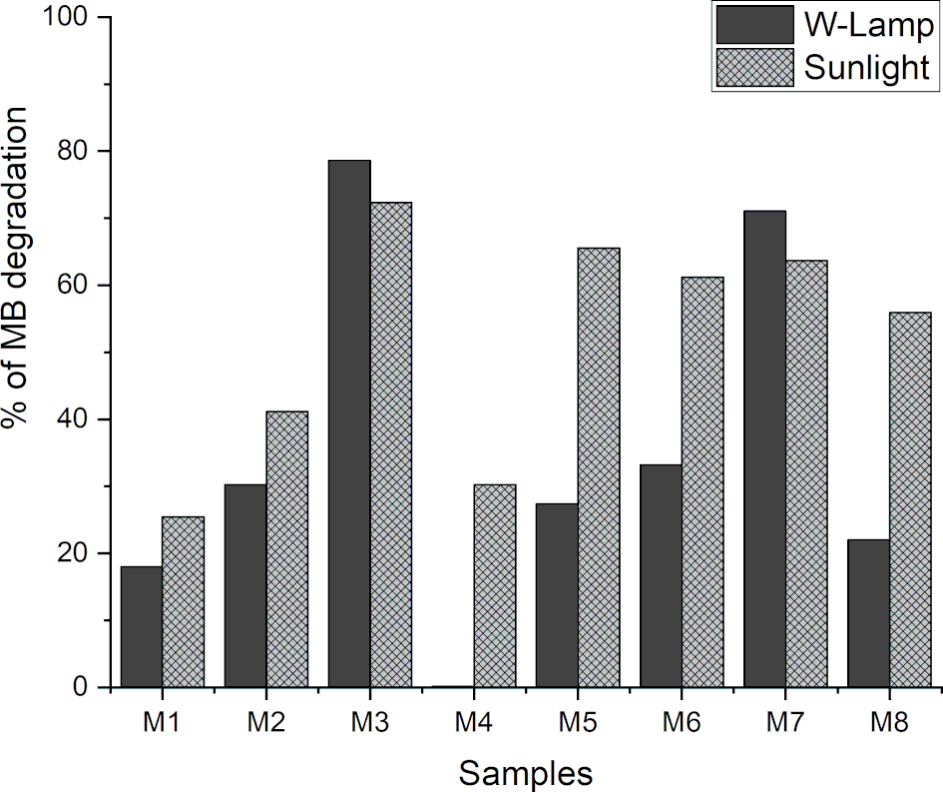
Percentage of MB degradation presented by CQDs to be used as catalysts in the degradation of MB dye. a) Solid bars: incandescent light, b) Hatched bars: solar light.

Finally, in Figure 10, the percentages of degradation achieved for each of the catalysts under the irradiation of the two types of light are presented. These results confirm that sample M3 synthesized from grape pomace exhibits the highest level of degradation, reaching 78% under incandescent light and 72% under sunlight. Thus, it demonstrates superior activity compared to other reported CQDs used as catalysts, such as the nanomaterials obtained from uchuva and luminol.

The comparison of MB degradation from CQDs shows that most samples effectively degraded MB. Notably, samples M4 and M8, synthesized without certain compounds, exhibit limited activity under incandescent light but improve significantly under sunlight. Additionally, smaller-sized samples demonstrate higher catalytic activity, with sample M3 from grape pomace exhibiting superior performance compared to that of other reported CQDs.

## Conclusion

The successful synthesis of luminescent and stable carbon quantum dots (CQDs) via the hydrothermal method using biomass as a precursor was achieved. We found that optimized synthesis parameters led to CQDs with diverse chemical characteristics. Notably, samples derived from grape pomace and watermelon peels synthesized with nitric acid, exhibited superior catalytic activity in methylene blue degradation, along with enhanced luminescence and stability compared to those synthesized with urea. It was also found that heteroatoms in the CQDs structure play an important role in the photocatalytic activity of the CQDs as well as in the upconversion photoluminescent behavior.

Our investigation also revealed that solar light was more effective than incandescent light in catalyzing reactions, and smaller-sized CQDs (1–10 nm) displayed higher catalytic activity, particularly evident in grape-pomace-derived samples. The superior performance of certain samples highlights the critical role of synthesis parameters and biomass sources in tailoring CQD properties for advanced photocatalytic applications. Furthermore, the assessment of luminescence activity unveiled the potential of CQDs for biomedical imaging, particularly with upconversion luminescence. This presents opportunities for targeted cell identification and drug delivery.

This study underscores the efficacy of the top-down hydrothermal method for CQD production, offering insights for tailored applications and emphasizing the potential of biomass-derived nanomaterials in environmental remediation and biomedicine, paving the way for the development of sustainable and effective technologies.

## Data Availability

The data that supports the findings of this study is available from the corresponding author upon reasonable request.
